# COVID-19-Induced Hypercoagulability: A Case Report

**DOI:** 10.7759/cureus.22155

**Published:** 2022-02-12

**Authors:** Bailey Sperry, Jenee Joseph, Benjamin Yglesias

**Affiliations:** 1 Psychiatry, American University of Antigua, College of Medicine, New York, USA; 2 Internal Medicine, American University of Antigua, St. John's, ATG; 3 Surgery, Trumbull Regional Medical Center, Warren, USA

**Keywords:** case report, atherothrombotic, dic, micro-embolism, hypercoagulability, covid-19

## Abstract

We report a case of atherothrombotic microembolism in a 53-year-old male diagnosed with coronavirus disease 2019 (COVID-19) prior to hospital admission. Upon admission, Day 9 after diagnosis, he presented with COVID-19 pneumonia and mottling of the lower extremities. The patient was treated with anticoagulation therapy. The lower extremity angiogram showed a patent posterior tibial artery and a patent peroneal artery. Despite initial anticoagulation therapy, toe and transmetatarsal amputations were required. However, a below-the-knee amputation was subsequently required due to continued worsening and extension of mottling. Unfortunately, the patient ultimately expired from cardiopulmonary arrest before any other surgical intervention could be done.

## Introduction

The global pandemic of severe acute respiratory syndrome (SARS), known now to cause coronavirus disease 2019 (COVID-19) pneumonia, hit the United States with its first confirmed case in January 2020 [1]. This virus, severe acute respiratory syndrome coronavirus 2 (SARS-CoV-2), has been well-documented to impact the respiratory tract, most notably in the form of pneumonia. However, multiple organ systems and homeostatic instability have increasingly become involved and are no longer limited to the respiratory tract. One process that has been extensively seen and documented is that of hypercoagulable states and coagulopathies. Of note, these hypercoagulable states have been linked to a worse prognosis especially when seen in critically ill patients [2]. This case report describes a COVID-19 patient who developed arterial microvascular thromboembolism.

## Case presentation

A 53-year-old Caucasian male, with a past medical history of hypertension, chronic renal disease, hepatitis A, and hospitalization for pancreatitis with elevated liver function tests (LFTs) and acute hepatitis, presented to the emergency department with shortness of breath, confusion, and some mottling of the lower extremities after previously being diagnosed nine days prior with COVID-19. Initial labs performed in the emergency department showed an elevated leukocyte count at 16,400/mm^3^ (normal: 4500-11,000/mm^3^), hemoglobin of 17.9 g/dL (normal: 13.5-17.5 g/dL), platelet count of 292,000/mm^3^ (normal: 150,000-400,000/mm^3^), lactate dehydrogenase (LDH) of 995 U/L (normal: 45-90 U/L), blood urea nitrogen (BUN) of 106 mg/dL (normal: 7-18 mg/dL), and creatinine of 3.24 mg/dL (normal: 0.6-1.2 mg/dL). The patient was initially started on vitamin C, zinc, thiamine, dexamethasone, and heparin for anticoagulation prophylaxis, which has been a common treatment method for patients with COVID-19 pneumonia. As the patient was treated for his initial diagnosis, the condition of his lower extremities worsened by Day 6. Both extremities had diminished pulses and were cool to touch from the mid-calf to toes with mottling confined to the toes. The left lower extremity was significantly worse than the right. Vascular surgery was consulted after it was noted to have a mottled/cyanotic left foot and toes with no improvement despite pharmacologic treatment. Surgery noted a palpable pulse overnight except for dorsalis pedis, and it was determined at that time that surgical intervention was not needed. Subsequently, he was placed on a heparin drip after arterial ultrasound duplex showed microemboli in the toes bilaterally. Mottling continued to expand to the heels. The condition of the lower extremities continued to worsen, requiring podiatric surgical intervention 22 days after initial admission.

**Figure 1:**
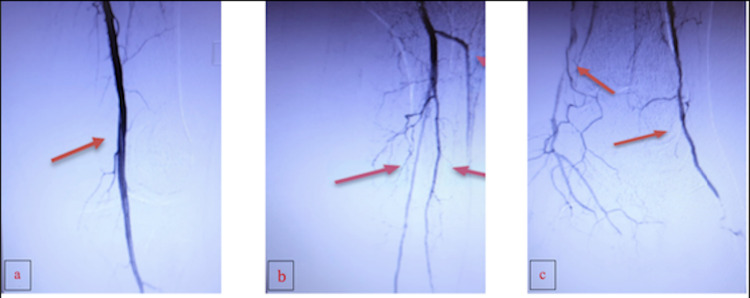
Angiogram of the left lower extremity Panel a shows adequate flow through the superficial femoral artery to the popliteal artery. Panel b shows an adequate flow from the popliteal artery to the trifurcation. Panel c shows adequate flow through the deep perineal artery and posterior tibial artery.

This patient developed acute-on-chronic renal disease, secondary to the onset of COVID-19 pneumonia, which limited the use of a contrast dye for imaging, specifically on the first presentation in the emergency department, and a pulmonary function test (PFT) was ordered to rule out other possible sources of acute respiratory failure. A computed tomography angiography (CTA) with runoff was performed and showed multifocal, relatively severe air-space disease, patent lower extremity arterial system bilaterally, with three-vessel run-off and fecal retention. Vascular surgery performed a left lower extremity angiogram that revealed a patent superficial femoral artery (SFA) (Figure 1a), a patent popliteal artery (Figure 1b), a patent posterior tibial artery (PTA), and a patent peroneal artery proximally (Figure 1c) as shown by the arrows in each image. At that time, a 3 mm balloon in the PTA and angioplasty was performed in two segments as well as in the popliteal artery. An interval angiogram revealed significant improvement of blood flow in the anterior tibial artery (ATA) and PTA. Small extravasation of contrast was noticed in the branches of the pedal arch vessels.

Due to the patient’s hypercoagulable state, the patient was started on enoxaparin 100. The patient was then switched to heparin 25000 in 250mL but was placed back on enoxaparin when noted to have a slight decrease in platelet count (261,000/mm^3^ to 133,000/mm^3^) two days after starting heparin. The patient was returned to the heparin drip after two days when it was noted to have continued decrease in platelet count (133,000/mm^3^ to 97,000/mm^3^) on enoxaparin and the lower extremity mottling continued to worsen despite heparin therapy and a CTA with good runoff. When the patient was found to be heparin-induced thrombocytopenia (HIT) positive via the Hep pf4 Ab screen, the patient was started on argatroban 250 mg. Further studies for HIT were run and serotonin release assay unfractionated heparin (SRA UFH) low dose returned <1 and SRA UFH high dose returned <1.

Prior to the patient’s demise, he presented with an activated partial thromboplastin time (aPTT) >139 (normal: 25 - 36 secs), prothrombin time (PT) of >90 (normal: 10 - 13 secs), and international normalized ratio (INR) of >8 (normal: 1). Other laboratory findings, including a platelet count of 100 (normal: 150,000 - 450,000), hemoglobin of 7.6 (normal: 12 - 15 g/dL) with a hematocrit of 25.1 (normal: 36 - 44 percent), and a fibrinogen level of 380 (normal: 130 - 330 mg/dL), were consistent with the International Society of Thrombosis and Hemostasis (ISTH) criteria for disseminated intravascular coagulation (DIC). The patient’s respiratory status continued to deteriorate, and he subsequently expired before any other intervention could be done. The 53-year-old Caucasian male had a 27-day total length of stay in the hospital before cardiopulmonary arrest.

## Discussion

SARS-CoV-2 has been shown to produce a wide range of features from dyspnea, fever, headache, and fatigue to severe viral pneumonia [3]. However, to date, increasing reports of hypercoagulability in COVID-19 patients have been documented. Previously, it is already known that infections can be associated with an increased risk of thrombosis. This may be due to venous stasis (such as this critically ill patient), immobilization, activation of platelets due to endothelial damage, shifts in the coagulation cascade, or vascular endothelial dysfunction (such as chronic inflammation) [4]. Although there is a correlation between COVID-19 and hypercoagulability, not much is known about the mechanism [5]. One possible mechanism being flouted is an increased inflammatory response, which has resulted in hypercoagulable states [2]. It is thought that the damage to the endothelial lining leads to a wave of events, including, but not limited to, activation of the coagulation cascade and release of pro-inflammatory cytokines. This, is turn, inhibits the anti-thrombotic pathways and may be a source of hypercoagulability as seen here.

Many research studies have shown that C-reactive protein (CRP), erythrocyte sedimentation rate (ESR), serum ferritin, and interleukin-6 (IL-6) have been found to be elevated in patients with COVID-19 pneumonia. Many patients have also been shown to have increased levels of D-dimer, lactate dehydrogenase (LDH), and prolonged prothrombin time [1]. Additionally, these biomarkers may be associated with COVID-19 coagulopathy with complement and platelet activation along with a cytokine storm activation that contributes to thrombus formation [5-6].

In this case report, the patient with a confirmed diagnosis of COVID-19 pneumonia, who also presented with mottling of the lower extremities, received prophylactic anticoagulation upon his hospital admission. Despite prophylactic treatment, the mottling of his lower extremities continued to worsen. Along with clinical suspicion and clinical biomarkers, a hypercoagulable state within this patient was suggested. An ultrasound duplex showed microembolism in the toes while a CTA of the lower extremities showed patent arterial flow. Here, the clinical presentation of this patient is consistent with a retrospective study done by Zhang. The study indicated that critically ill COVID-19 patients could present with acro-ischemia (digital microvascular ischemia) [7-8]. Moreover, despite not having a clear source of embolization, studies have shown that his preexisting comorbidity of hypertension increases the risk of microemboli during COVID-19 illness [8-9].

It has been shown that critically ill patients with COVID-19 pneumonia have been associated with increased inflammatory response, elevated D-dimer, and abnormal coagulation parameters consistent with systemic activation of hemostasis. These parameters may present similarly to disseminated intravascular coagulation (DIC) and sepsis-induced coagulopathy, which were also visible in this patient [10]. As seen in this patient, the signs and symptoms of a thrombotic state, such as digital necrosis are related to perimortem DIC and gangrene [11].

Although the patient ultimately met his demise, studies support the benefit and use of anticoagulation in critically ill patients, as benefits outweigh the risk. In a retrospective case review of all consecutive adult patients admitted to eight French ICUs for severe laboratory-confirmed COVID-19 pneumonia, it was shown that the use of high-dose prophylactic anticoagulation (such as low-molecular-weight heparin (LMWH) 40 mg once daily or unfractionated heparin (UFH) 5000 units subcutaneously every eight hours) is associated with a reduction in thrombotic risk without increasing the risk of bleeding [12-13]. Standard anticoagulation prophylaxis has been shown to be efficacious but has not been shown to reduce mortality rates. Adverse effects with anticoagulation therapy, such as thrombocytopenia, should be monitored by clinicians with daily labs and examination of the patient for signs of bleeding. Further studies for alternative treatment methods should be investigated, which provide both mortality and morbidity benefits. The risks and benefits of anticoagulation therapy should be discussed with the patient as well as other healthcare providers.

## Conclusions

The case shows that COVID-19 can activate a hypercoagulable state by activating numerous pathways, including complement and chronic inflammation. It also highlights the manifestation and worsening of distal arterial microemboli although the patient received anticoagulation therapy and no history suggesting a hypercoagulable state. Furthermore, the study demonstrates the presence of hypercoagulability in critically ill COVID-19 patients may also prove to be fatal.
